# The impact of preventive health behaviour and social factors on visits to the doctor

**DOI:** 10.1186/2045-4015-3-41

**Published:** 2014-12-18

**Authors:** Gregory Yom Din, Zinaida Zugman, Alla Khashper

**Affiliations:** The Open University of Israel, Raanana, Israel, Faculty of Exact Sciences, Tel-Aviv University, Tel-Aviv, Israel; Kazrin, Israel; McGill University Health Center, Montreal, Canada

**Keywords:** Visits to the doctor, Universal health care, Preventive health behavior, Ordered probit, Endogeneity

## Abstract

**Background:**

The aim of this study is to examine the joint impact of preventive health behavior (PHB) and social and demographic factors on the utilization of primary and secondary medical care under a universal health care system, as measured by visits to the doctor, who were categorized as either a General Practitioner (GP) or Specialist Doctor (SD).

**Methods:**

An ordered probit model was utilized to analyze data obtained from the 2009 Israeli National Health Survey. The problem of endogeneity between PHB factors and visits to GP was approached using the two-stage residuals inclusion and instrumental variables method.

**Results:**

We found a positive effect of PHB on visits to the doctor while the addition of the PHB factors to the independent variables resulted in important changes in explaining visits to GP (in values of the estimates, in their sign, and in their statistical significance), and only in slight changes for visits to SD. A 1% increase in PHB factors results in increasing the probability to visit General Practitioner in the last year in 0.6%. The following variables were identified as significant in explaining frequency of visits to the doctor: PHB, socio-economic status (pro-poor for visits to GP, pro-rich for visits to SD), location (for visits to SD), gender, age (age 60 or greater being a negative factor for visits to GP and a positive factor for visits to SD), chronic diseases, and marital status (being married was a negative factor for visits to GP and a positive factor for visits to SD).

**Conclusions:**

There is a need for allowing for endogeneity in examining the impact of PHB, social and demographic factors on visits to GP in a population under universal health insurance.

For disadvantaged populations with low SES and those living in peripheral districts, the value of *IndPrev* is lower than for populations with high *SES* and living in the center of the country. Examining the impact of these factors, significant differences in the importance and sometimes even in the sign of their influence on visits to different categories of doctors - GP and SD, are found.

**Electronic supplementary material:**

The online version of this article (doi:10.1186/2045-4015-3-41) contains supplementary material, which is available to authorized users.

## Background

When do people visit the doctor? To answer this question, an important one for health policy planning, researchers analyze characteristics of the population under study (potential patients), medical institutions, and social structure as they relate to health care issues [1]. It is necessary to categorize doctors by specialization – there are characteristics of a population whose variations can influence, in opposite directions, the probability of visiting General Practitioner (GP) against Specialist Doctor (SD). For example, in studying migrants’ health, authors cited income as a characteristic positively affecting visits to GP, and negatively affecting visits to SD [2].

Preventive health behavior (PHB), or “activity undertaken by a person who believes himself to be healthy for the purpose of preventing disease” [3], is one of the characteristics of a population which affects the frequency of visits to a doctor. Pender suggested that PHB and health-promoting behavior (e.g. physical activities) complement one another [4], and the term PHB is used in this study to refer to these two interrelated dimensions of a healthy lifestyle. A number of recent studies report the influence of various PHB factors on medical care utilization including smoking [5], obesity [6], influenza vaccination [7], physical activity [8] or combination of PHB factors [9]. Similar studies were undertaken in Israel: on smoking [10], on physical activity [11], on influenza vaccination [12], and on mammography [13].

The aim of this study is to examine the joint impact of PHB and social and demographic factors of individuals on the utilization of primary and secondary medical care, as measured by visits to both GP and SD doctors. In the case of Israel, it is assumed that due to universal health insurance, and the fact that more than half GPs are not paid through a fee-for-service system, the frequency of visits to the doctor are not greatly affected by variations in medical institutions and social structure.

Analysis of disadvantaged populations and their social-demographic characteristics is important for this study. For these populations, motives for engaging in PHB may be weaker due to low socio-economic status (SES) and lower income [14, 15], advanced age [16], gender [17], residence in a disadvantaged neighborhood [18], or lack of education [19]. This weaker motivation can be explained by lack of social support [20], lack of access to facilities for engaging in physical activity or a healthy diet [21], or lack of awareness and understanding health care issues [22]. A similar relationship is shown by many Israeli studies: for older populations [23], for residents in the periphery [24], for women [25], and for people with lower SES [26].

The problem of the inequity in PHB is reported for several countries with universal health insurance: in European countries [16], Australia [27], and Japan [28].

In the current study, we treat “visits to the doctor” as a discrete ordered variable and utilize an ordered probit model to measure the joint impact and marginal effects of PHB and disadvantage factors on frequency of visits to the doctor. Concurrently, PHB can be itself influenced by medical care utilization, for example as a result of visiting a doctor, which leads to the problem of endogeneity between medical care utilization and PHB.

Authors [29] note that visits to GP can significantly influence a patient’s lifestyle by identifying unhealthy behavior. In another similar context, researchers conclude that after allowing for endogeneity, an increase in visits to a GP has a significant positive effect on self-reported health for individuals [30]. However, in most studies which analyze the involvement of PHB in explaining frequency of visits to the doctor, the potential endogeneity of PHB factors was not controlled for.

We addressed this problem to avoid biased estimates in statistical modeling by using the instrumental variables method. Regarding the influence of disadvantage factors, we expected to find pro-poor inequality in visits to GP and pro-rich inequality to visits to SD, as has been previously reported in many studies of OECD countries that provide universal health insurance [31, 32]. We also expected to identify additional characteristics which can influence positively or negatively on probabilities of visiting GP or SD, and to find evidence for the above-mentioned statements that the motives for engaging in PHB may be weaker for disadvantaged populations.

## Methods

### Data

The data for this study was obtained from the 2009 Israeli National Health Survey, whose questionnaire matched OECD data requirements. The survey was given in Hebrew, English, Russian, and Arabic as required for different population groups. The data was collected by interview during visits to 8,713 households, covering 28,968 individuals in total. For every respondent, his or her relationship to the head of household was noted.

The summary of the respondents’ disadvantage and PHB factors is presented in Table 1. On the whole, the samples were large enough for statistical tests and inference.

**Table 1:** **2009 Israeli national health survey: disadvantage and PHB factors of respondents**

Characteristics	Respondents	%
Total respondents	28968	100%
Low SES	10129	35%
Live in periphery	4572	16%
Older population (age ≥60)	7084	24%
Physical activity		
No physical activity	23312	80.5%
1-2 days/week	1810	6.3%
3-4 days/week	1977	6.8%
5 days/week	383	1.3%
More than 5 days/week	1486	5.1%
Smoking		
No smoking	25230	87.1%
1-10 cigarettes/day	1511	5.2%
11-20 cigarettes/day	1424	4.9%
More than 21 cigarettes/day	803	2.8%
Received influenza vaccination	3670	12.7%
Underwent mammography (women only)	3929	26.5%
Have supplemental insurance	21787	75.2%

In this survey, GP was considered to have an expanded definition which included family doctors, pediatricians and internal physicians. We used the number of visits to GP as a measure for primary medical care utilization. Specialist doctors (SD) included gynecologists, orthopedists, ear-nose-throat specialists, ophthalmologists, cardiologists, surgeons, urologists, neurologists, pulmonologists, psychiatrists, and other medical practitioners (unlike some other countries, in Israel general practitioners do not perform gynecological examination and routine pregnancy care). The number of visits to SD was used as a measure for secondary medical care utilization.

### The model

#### Model versions and variables

Various versions of the proposed ordered probit model differed in (a) the dependent variable (visits to GP and visits to SD), and (b) the independent variables that included or did not include PHB factors. Additionally, in the model which utilized the variable of visits to GP, the endogeneity problem was addressed.

The following data was used as dependent variables:

*VisGP* – number of visits to a GP over the two weeks before survey participation; and

*VisSD* – number of visits to a SD over the two weeks before survey participation.

For independent variables, three groups were examined: 1) variables related to PHB; 2) variables associated with factors endemic to disadvantaged populations; and 3) other social, demographic, and health related variables.

First, the following PHB variables were examined: smoking, physical activity, influenza vaccination, mammography screening, and purchase of supplemental insurance. Mammography was accounted for women aged 50–74 for whom this test is provided free of charge every two years due to the National Health Insurance Law (1995). Therefore, taking mammography has been treated as a health preventive factor for this group. Then, the following disadvantage factors were examined: SES (low, middle, or high level), age (below or over 60 years of age), and location (peripheral, intermediate, or central regions). The following social, demographic, and health related factors were utilized: gender, diagnosis (or not) of at least one chronic disease, employment (full- or part-time) versus unemployment, religion, housing density (number of persons per room), and marital status. Not all of the examined factors were included directly in the final versions of the model. Several of them were found insignificant in all versions of the model, and others were used in the form of an index of PHB factors.

A summary of data examined as dependent and independent variables in various versions of the models is shown in Table 2.

**Table 2:** **Variables examined for use in the ordered probit model**

Name	Notation	Description in the survey	Values
Dependent variables			
Primary medical care	*VisGP*	visits to GP in the last two weeks	0 = no visits, 1 = one visit, 2 = more than one visit
Secondary medical care	*VisSD*	visits to SD in the last two weeks	0 = no visits, 1 = one visit, 2 = more than one visit
Independent variables associated with disadvantaged populations			
Social-economic status	*SES*	See 2.2.1	1 = low, 2 = middle, 3 = high
Location	*Loc*	District of residence	1 = peripheral, 2 = intermediate, 3 = central
Age	*Age*	Age group	0 = age < 60, 1 = age ≥ 60
Independent variables associated with social, demographic and health factors			
Gender	*Gen*	Sex	0 = male, 1 = female
Religion	*Rel*	Registered religion of head of household	0 = Jewish, 1 = other
Chronic diseases	*Chron*	One chronic disease at least	0 = no, 1 = yes
Employment characteristics	*WeelkyLF*	Weekly labor force characteristics	0 = unemployed, 1 = full- or part-time employment
Density	*Dens*	Housing density	14 ordered categories, based on the number of persons to a room
Marital	*Mar*	Marital status	0 = married, 1 = unmarried
The father’s origin	*FatherCont*	Father's continent of birth	0 = Europe, America, Israel, 1 = other
Independent variables associated with PHB			
Bodily activity	*BodyAct*	Amount of weekly exercise	1 = no physical activity, 2 = 1–2 days/week, 3 = 3–4 days/week, 4 = 5 days/week, 5 = more than 5 days/week
Smoking	*Smoke*	Number of cigarettes a day	1 = no smoking, 2 = 1–10 a day, 3 = 11–20 a day, 4 = more than 21 a day
Flu vaccination	*Flu*	Vaccination against influenza	0 = no, 1 = yes
Mammography	*Mamm*	Mammography test, for women of age 50-74	0 = no, 1 = yes
Supplemental insurance	*SupIns*	Purchase of supplemental health insurance	0 = no, 1 = yes

#### Calculating SES index

The following data was used for calculation of SES: occupation, years of schooling, socio-economic cluster, and size of family residence (number of rooms). The socio-economic clusters (SEC) used by the Israel Central Bureau of Statistics to classify municipalities or regional councils are created using an average of per-capita income, percentage of vehicle owners, and other relevant characteristics [33]. These area-based measures are used by researchers to monitor and assess socio-economic inequalities in health care utilization [34].

The value of SES for every respondent was calculated as follows:
1

where *Occup* = occupation;

*Educ* = education;

*SEC* = socio-economic cluster; and

*rooms* = number of rooms in family residence of the respondent.

To prepare input data for the calculation of SES, the data from the survey (occupation, education, and SEC) were grouped and the coefficient *k* was determined so that each of the four data values for SES would receive values ranging from 1 to 5. These SES values were divided into three equal percentiles, corresponding to low, middle, and high levels of respondent SES [35].

#### Calculating index of PHB

The survey questionnaire included several questions related to PHB and health-promoting behavior. These answers were used to calculate the PHB index, *IndPrev*. This method has been used in previous studies where indices of PHB or of “good health practices” were suggested to be related to health care utilization by disadvantaged populations [36]. In our study, the index *IndPrev* was constructed as an equally weighted score given to the three (for women) or two (for men) items regarding PHB and lifestyle (Table 1).

First, we examined the following data for calculating *IndPrev*: *BodyAct*, *Smoke*, *Flu*, *Mamm*, and *SupIns* (Table 2). We tested these factors for internal consistency using Cronbach’s alpha, and found that *Smoke* and *SupIns* were not related enough to the other factors, and therefore were discarded.

The value of *IndPrev* for every respondent was calculated as follows:
2

For minors under 18 years the data of head of household were used for calculating *IndPrev*. This approach is based on the assumption that potential differences in child medical care consumption could be explained by differences in parental behavior [37].

#### Model specification

The formal description of the ordered probit model for doctor’s visits (GP or SD) was as follows:
3

where *i* is the number of the respondent;

y^*^ is an unobserved continuous variable that affects the observed outcomes of visits to the doctor (*VisGP* or *VisSD,* defined in Table 2), which can be interpreted as the propensity to visit the doctor);

**X**_*i*_ = (1, *x*_1_, …, *x*_*n*_)′ is a vector of explanatory variables (Table 2) of the individual;

**β** = (*β*_0_, *β*_1_, … *β*_*n*_)′ is a vector of ordered probit coefficients to be estimated; and

*ϵ*_*i*_ is a random normal distributed variable with zero expectation and standard deviation of *σ* equal to unity (a usual assumption in probit analysis).

While it is not possible to observe y^*^ in this model, the values of *VisGP* can be observed as follows:
4

Here *α*_0_, *α*_1_ provides the cutpoints for y^*^ which determine the observed value of *VisGP.* For the model utilizing *VisSD*, the relationship between *VisSD* and the unobserved variable y^*^ is determined in a similar manner.

The coefficient *β*_*j*_ shows the influence of the *j*^*th*^ independent variable on the probability that *VisGP* (or *VisSD*, depending on the model) receives the value from one of the end categories, either 0 or 2. If *β*_*j*_ is positive, the probability of receiving the value 0 declines and of receiving the value 2 increases. If *β*_*j*_ is negative, the opposite is the case [38].

For each of the models using *VisGP* and *VisSD*, the partial marginal effect of every continuous explanatory variable and for every observed value of visits to the doctor (0, 1, or 2) showed a change in the predicted probability of the dependent variable receiving this value, associated with one-unit incremental changes in the explanatory variable. In the case of discrete explanatory variables, instead of “one-unit change,” use “0 to 1 change,” or “1 to 2 change” (if the number of possible values is more than 1), and so on. The method used for effects calculation is detailed in Appendix.

As mentioned previously, endogeneity can exist between the PHB factors and *VisGP*. Visits to a GP can influence the patient’s PHB, particularly for the flu vaccination, physical activity and mammography variables. Flu vaccination is one of the most studied examples of primary preventive care [39]. The role of advice from primary care professionals in patients quitting smoking and adopting a more active lifestyle is discussed in [40]. In Israel, a biennial mammogram for women aged 50–74 is part of the preventative primary health care basket included in the universal health care system [41]. There does not seem to be reciprocal influence between visits to a SD and preventive health behavior such as a flu vaccination, exercise, or mammography. Therefore, we allowed for the potential endogeneity of the *IndPrev* variable in the visits to GP model only.

To cope with the potential endogeneity problem, a two-stage residuals inclusion (2SRI) method was used [42]. In the first stage of this method, the potentially endogenous variable *IndPrev* is regressed on the other independent variables and on a set of instrumental variables (IV). OLS regression is used at this stage. In the second stage, the first stage residuals are included as an additional regressor in the ordered probit model (3), and the modified ordered probit model for visits to the doctor (GP or SD) is as follows:
5

where  are the residuals obtained from estimating (3);

**γ** is a probit coefficients vector for the explanatory variables;

*δ* is a probit coefficient of the residuals from the first stage; and

*η*_*i*_ is the error term. The other symbols are the same as in (3).

If the estimate of *δ* is significant, the exogeneity hypothesis for *IndPrev* is rejected and the instrumentation is accepted as plausible, a method applied in many studies [43]. After several trials, the following IV variables for the first stage were selected: *Dens* and *FatherCont*. Each of the variables was negatively correlated with *IndPrev* (the direction of changes in these variables is clear from their description in Table 2).

## Results

The mean values of *IndPrev* were calculated for various groups of disadvantaged populations.

For age < 60, *IndPrev* = 0.104 ± 0.002, for older population of age ≥ 60, *IndPrev* = 0.384 ± 0.009.

For low *SES*, *IndPrev* = 0.116 ± 0.004, for middle *SES IndPrev* = 0.154 ± 0.005, and for high *SES IndPrev* = 0.171 ± 0.005.

For location in the periphery, *IndPrev* = 0.133 ± 0.007, in an intermediate location *IndPrev* = 0.130 ± 0.005, and in the center *IndPrev* = 0.159 ± 0.004.

For disadvantaged populations with low SES, the value of *IndPrev* is lower than for populations with middle or high *SES*, and for those living in peripheral districts, *IndPrev* is lower than for those living in the center of the country. For older populations, the *IndPrev* value is some 3.7 times greater than for those under 60 years of age. All of these comparisons are highly significant.

The ordered probit models (Tables 3 and 4) were estimated using IBM Statistics SPSS 20 software (Additional file 1), with the partial marginal effects calculated using MS Excel worksheets (Tables 5 and 6).

**Table 3:** **Estimates, their 95% confidence intervals and significance for the ordered probit model “visits to the GP”**

Variables	Without ***IndPrev***		With ***IndPrev***			
	Without IV		Without IV		2SRI	
	Estimates	Significance	Estimates	Significance	Estimates	Significance
[*VisGP* = 0]	1.325 ± 0.050	0.000	1.344 ± 0.050	0.000	1.489 ± 0.054	0.000
[*VisGP* = 1]	2.093 ± 0.051	0.000	2.114 ± 0.051	0.000	2.261 ± 0.056	0.000
*IndPrev*			0.345 ± 0.041	0.000	3.037 ± 0.399	0.000
*SES*	-0.065 ± 0.013	0.000	-0.074 ± 0.013	0.000	-0.135 ± 0.016	0.000
*Loc*	0.002 ± 0.013	0.889	0.003 ± 0.013	0.841	0.007 ± 0.014	0.605
*Age*	0.414 ± 0.027	0.000	0.342 ± 0.028	0.000	-0.222 ± 0.087	0.011
*Gen*	0.093 ± 0.019	0.000	0.083 ± 0.019	0.000	-0.021 ± 0.021	0.323
*Chron*	0.529 ± 0.024	0.000	0.499 ± 0.025	0.000	0.259 ± 0.043	0.000
*SupIns*	0.096 ± 0.024	0.000	0.081 ± 0.024	0.001	-0.022 ± 0.028	0.433
*Mar*	0.016 ± 0.020	0.430	0.032 ± 0.020	0.107	0.142 ± 0.026	0.000
*Rel*	-0.019 ± 0.028	0.487	-0.014 ± 0.028	0.610	0.023 ± 0.028	0.419
Residuals from the 1st stage					-2.727 ± 0.402	0.000

**Table 4:** **Estimates, their 95% confidence intervals and significance for the ordered probit model “visits to the SD”**

Variables	Without IndPrev		With IndPrev	
	Estimates	Significance	Estimates	Significance
[*VisSD* = 0]	1.924 ± 0.065	0.000	1.939 ± 0.065	0.000
[*VisSD* = 1]	2.609 ± 0.067	0.000	2.624 ± 0.067	0.000
*IndPrev*			0.265 ± 0.052	0.000
*SES*	0.064 ± 0.017	0.000	0.057 ± 0.017	0.001
*Loc*	0.028 ± 0.018	0.116	0.030 ± 0.018	0.093
*Age*	0.247 ± 0.033	0.000	0.186 ± 0.035	0.000
*Gen*	0.188 ± 0.024	0.000	0.185 ± 0.024	0.000
*Chron*	0.439 ± 0.030	0.000	0.415 ± 0.030	0.000
*SupIns*	0.062 ± 0.032	0.048	0.050 ± 0.032	0.111
*Mar*	-0.239 ± 0.025	0.000	-0.231 ± 0.025	0.000
*Rel*	-0.080 ± 0.038	0.036	-0.074 ± 0.038	0.052

**Table 5:** **Partial marginal effects for the ordered probit model “visits to GP”**

Variables	Version (c) with IndPrev and IV		
	VisGP = 0	VisGP = 1	VisGP = 2
*IndPrev*	-0.588	0.416	0.173
*SES*	0.026	-0.019	-0.008
*Loc*	-0.001	0.001	0.000
*Age*	0.043	-0.030	-0.013
*Gen*	-0.004	0.003	0.001
*Chron*	-0.050	0.036	0.015
*SupIns*	0.004	-0.003	-0.001
*Mar*	-0.028	0.019	0.008
*Rel*	-0.004	0.003	0.001

**Table 6:** **Partial marginal effects for the ordered probit model “visits to SD”**

Variables	Version (b) with IndPrev		
	VisSD = 0	VisSD = 1	VisSD = 2
*IndPrev*	-0.028	0.020	0.007
*SES*	-0.006	0.004	0.002
*Loc*	-0.003	0.002	0.001
*Age*	-0.019	0.014	0.005
*Gen*	-0.019	0.014	0.005
*Chron*	-0.043	0.032	0.011
*SupIns*	-0.005	0.004	0.001
*Mar*	0.024	-0.018	-0.006
*Rel*	0.008	-0.006	-0.002

Three versions of the visits to GP model were estimated: (a) without *IndPrev*, (b) with *IndPrev* and without IV, and (c) with *IndPrev* and with IV. The variable *IndPrev* was highly significant in versions (b) and (c), and for the latter, the residuals estimate from the 1st stage was significant. Therefore, the possibility of exogeneity for *IndPrev* was rejected and version (a) was compared with the version (c).

For the variable *SES*, the negative sign of the estimate for both versions (a) and (c) showed that higher values of this variable result in an increased probability that *VisGP* = 0 and a decline in the probability that *VisGP* = 2. Exactly the opposite follows from the positive signs of the estimates for the dummies *Chron* (a 1 value indicating presence of at least one chronic disease) and *Mar* (a 1 value indicating unmarried status). For all three variables, *SES*, *Chron*, and *Mar*, the estimates for version (c) were significantly different from those in (a), as their confidence intervals show. The dummy *Age* (if 1 then age ≥ 60) estimate changed the sign – its negative sign in version (c) shows that in older populations the probability of *VisGP* = 0 increases and the probability of *VisGP* = 2 declines. The estimates for *Gen* and *SupIns* (gender and supplemental health insurance) became non-significant in the version (c), and for *Rel* – non-significant in all versions (Table 3).

Two versions of the visits to SD model were estimated: (a) without *IndPrev*, and (b), with *IndPrev* (instrumentation was not employed in this model). Like in the visits to GP model, the added variable *IndPrev* was highly significant in version (b). The estimates for all other variables were not significantly different from those in version (a), as seen from an examination of their confidence intervals. Most estimates were significant at a 5% level, except for *Loc* and *SupIns* in version (b) (Table 4).

Examination of the partial marginal effects for both models garnered additional information on changes in the probabilities of the subsample level dependent variables when the number of visits equaled 0, 1, or more than 1. For the model for visits to the GP (Table 5), the signs of the effects for the subsample *VisGP* = 0 were the opposite of the signs of the estimates for the same version of the model (Table 3, the next to last column). For example, for the variable *SES*, as mentioned previously, the negative sign of the estimate (-0.135) shows that an increase in this variable results in an increased probability that *VisGP* = 0. In line with this, the values of the partial marginal effects (0.026, -0.019, -0.008) show that a one unit increase in *SES* results in increasing the probability that *VisGP* = 0 in 2.6%, in decreasing the probability that *VisGP* = 1 in 1.9%, and in decreasing the probability that *VisGP* = 2 in 0.8%. The sum of the marginal effects of *SES* calculated for different values of *VisGP* equals zero (allowing for rounding error). This property holds for each explanatory variable (Appendix). For *IndPrev*, a 1% increase in this index results in increasing the probability that the person visited GP in the last year, in 0.6% approximately (the partial marginal effect is -0.588 in Table 5).

In a similar manner, the partial marginal effects (Table 6) were analyzed and compared to the estimates (Table 4) for the model of visits to SD. The effect of *IndPrev* on visits to SD (Table 6) is much less than the effect of the same variable on visits to the GP (Table 5).

## Discussion

This study examined factors explaining the frequency of visits to a doctor for citizens of a country with universal health insurance. The study used data from a national health care survey, and indices calculated from its data, for ordered probit modeling of factors explaining visits to both GP and SD doctors. For both versions of the model used, the same explanatory variables were explored twice, once by taking into account the respondents’ PHB as expressed by the index *IndPrev*, and once by leaving *IndPrev* out of the model. This variable was highly significant in both versions, but the influence of its addition on the model estimates was found to be different: there were important changes for visits to GP (in values of the estimates, in their sign, and in their statistical significance) by adding *IndPrev*, and only slight changes for visits to SD.

In the version of visits to GP, the instrumental variables – housing density (*Dens*) and father's continent of birth (*FatherCont*) – were selected to approach the problem of endogeneity between visits to GP and *IndPrev*. By using the 2SRI method, the exogeneity hypothesis for *IndPrev* was rejected and the instrumentation was accepted as plausible.

The following variables were identified as significant for explaining frequency of visits to the doctor:

- Preventive health behavior (PHB) – positive influence of more health preventive behaviors on the probability to visit GP and SD (henceforth “positive” or accordingly “negative”);
- Socio-economic status (SES) – negative for visits to GP (pro-poor) and positive for visits to SD (pro-rich inequality);
- Location, gender, and supplemental health insurance, for visits to SD only – positive for central districts (9% level of significance), for females, and for those who have supplemental insurance (5-11% level of significance);
- Age – age of 60 or greater, negative for visits to GP and positive for visits to SD;
- One chronic disease at least – positive; and
- Marital status – if married, negative for visits to GP and positive for visits to SD.

Many findings in this article are in line with previous studies which researched public health under a universal health care system. Our study reveals that the probability to visit GP and SD is positively influenced by PHB which is consistent with results [44, 29].

Furthermore, we demonstrate pro-poor inequality in visits to GP and pro-rich inequality in visits to SD that are concordant with the findings of other studies conducted in OECD countries where universal health insurance is provided [31, 32]. In Canada, patients of lower SES were found to have had significantly more primary care visits, while the differences in utilization of specialty services were less pronounced and often not statistically significant [45]. In Israel, the income-related inequality in primary care is pro-poor, and in secondary physicians’ services is pro-rich [46].

Our results show the positive influence of location in central districts on the frequency of visits to SD. Similar results were found in [16], where the authors note that medical care becomes more accessible in areas with higher physician availability, leading to high levels of use.

This study identified age as a factor that can influence the probability of visiting a GP or SD in opposite directions, ceteris paribus, particularly when controlled for PHB and at least one chronic disease factors. This conclusion is similar to the report [47] where it was concluded that given the high morbidity burden between elder populations, higher use of specialist physicians is found but not higher use of primary care physicians.

Our result that men visited SD less than women agrees with the studies [48] where it was found that in the USA men are less likely to visit doctors’ offices than women, and [49] where older females in 11 European studied countries evidenced higher levels of health care usage than males.

Our result that smoking was not related to other factors of PHB (particularly physical activity), made by looking for relevant variables for their inclusion in *IndPrev,* is in agreement with the results in [50] where physical activity was found unrelated to smoking for older persons in Israel.

The main limitations of this study are found in the choice of explanatory variables, because of the absence of data on health status and income of respondents in the National Health Survey questionnaire. These issues were addressed by using the “one chronic disease at least” question as a proxy for health status, and by using occupation and education level as proxies for income.

## Conclusion

The results enabled to examine the impact of PHB and social and demographic factors on visits to GP and SD doctors:

- There is a need to test endogeneity in examining the impact of PHB, social and demographic factors on visits to GP in a population under universal health insurance.
- For disadvantaged populations with low SES and those living in peripheral districts, the value of *IndPrev* is lower compared to populations with high *SES* and to those living in the center of the country.
- Examining the impact of the abovementioned factors, significant differences in the importance and sometimes even in the sign of their influence on visits to different categories of doctors - GP and SD, are found. As it was shown in this study, it relates, in particular, to *Loc*, *Gen*, *Rel*: non-significant / significant, and to *SES*, *Age*, *Mar*: negative / positive factors.

## Appendix

### Calculation of partial marginal effects

Parameter estimates from the ordered probit model (3) can be transformed to estimates of the partial marginal effects, which show the change in predicted probabilities of specific values of the dependent variable associated with changes in the explanatory variables. In the ordered probit model, a positive (negative) value of *β*_*k*_ means that higher values of *x*_*k*_ increase (decrease) the likelihood of higher values of the dependent variable *VisGP* (and *VisSD*, accordingly). Unlike the OLS regression, coefficients in the ordered probit model do not represent marginal changes in the dependent variable, given minor changes in the independent variables. The influence of a change in *x*_*k*_ on the probability *p* that *VisGP* or *VisSD* receive one of their possible values (0, 1, or 2) can be examined by taking the partial derivative of this probability, with respect to *x*_*k*_ ("partial marginal effect" of *x*_*k*_).

For the model with *VisGP*, and for each of continuous *x*_*k*_ the partial marginal effects *ME*_0,*k*_ (when *VisGP* = 0), *ME*_1,*k*_ (when *VisGP* = 1), *ME*_2,*k*_ (when *VisGP* = 2) is calculated as follows:
6

The *α*_0_,*α*_1_ are defined in (4), and *φ* denotes standard normal density function.

For discrete variables, for example for a binary (0, 1) variable *x*_*k*_, the effect of the zero-to-one discrete change for each value *j* of *VisGP* (*j* = 0, 1, or 2) is calculated simply as a difference between the probabilities of two events: *VisGP* = *j* for *x*_*k*_ = 1 and for *x*_*k*_ = 0. The effects of discrete changes for variables (1, 2, 3) are calculated in a similar manner.

In addition, for each of the explanatory variables the sum of its marginal effects calculated for different values of *VisGP*, equals 0. This follows immediately from (6) and from the described procedure for calculating marginal effects of the discrete variables.

For the model with *VisSD*, the partial marginal effects are calculated in a similar manner [51].

## Authors’ information

Gregory Yom Din, PhD, is a coordinator of the course of applied econometrics for MBA at the Open University of Israel and a researcher at the Faculty of Exact Sciences, Tel-Aviv University. His current research interests include econometric modelling of health care systems, particularly for disadvantaged populations. Zinaida Zugman, PhD, is a former researcher at Golan Research Institute, Katzrin, Israel and an independent consultant, with a special focus on development of mathematical models and simulation models for decision-making. Alla Khashper recently joined the department of Diagnostic Imaging at Soroka University Medical Center, Beer Sheva, Israel after completing her fellowship at McGill University, Montreal, Quebec. She is a lecturer at the medical school of Ben-Gurion University of the Negev, Beer-Sheva, Israel for local and international students.

## Electronic supplementary material

Additional file 1:: **SPSS programs.** (DOC 62 KB)
