# Supplementary material for: The impact of preventive health behaviour and social factors on visits to the doctor
Source: Isr J Health Policy Res. 2014 Dec 18;3:41. doi: 10.1186/2045-4015-3-41 (PMC4290136; doi:10.1186/2045-4015-3-41)
Supplement: Supplementary file 1 — Additional file 1:: SPSS programs. (DOC 62 KB) [file 13584_2014_140_MOESM1_ESM.doc]

*_____regression of visits to the doctor (separately – GP and SD)

*_____approaching potential endogeneity of Preventive Health Behavior (PHB)

*_____with a two-stage residuals inclusion method (2SRI)

GET FILE='*the full path and name of the file with the initial data*'

/KEEP all.

*_____Dependent variable VisGP_____.

*_____Dependent variable VisSD_____.

*_____Calculate dependent variables VisGP, VisSD for ordered probit_____.

DO IF (VisGP > 1).

COMPUTE VisGP = 2.

END IF.

DO IF (VisSD > 1).

COMPUTE VisSD = 2.

END IF.

*_____Calculate the index of PHB factors_____.

COMPUTE BodyAct_5 =(BodyAct-1)/4.

COMPUTE Smoke_5 =(5-Smoke)/4.

DO IF (Gen = 0).

COMPUTE IndPrev =(BodyAct_5+Flu)/2.

ELSE.

COMPUTE IndPrev =(BodyAct_5+Flu+Mamm)/3.

END IF.

*_____Calculate descriptive statistics_____.

DESCRIPTIVES VARIABLES = VisGP, VisSD, IndPrev, BodyAct, Smoke, Flu, Mamm, SupIns, Loc, Age, SES, Gen, Mar, Cont, Rel, Chron, weeklyLF, Dens, Res_2SRI.

CORRELATIONS VARIABLES = VisGP, VisSD, IndPrev, BodyAct, Smoke, Flu, Mamm, SupIns, Loc, Age, SES, Gen, Mar, Cont, Rel, Chron, weeklyLF, Dens, Res_2SRI.

*_____Calculate the Cronbach’s ALPHA_____.

RELIABILITY

/VARIABLES=Flu Mamm BodyAct Smoke_4

/MODEL=ALPHA

/STATISTICS=DESCRIPTIVE SCALE CORR

/SUMMARY=TOTAL.

*_____Running OLS regression for calculating residuals for 2SRI_____.

REGRESSION VARIABLES = IndPrev, SES, Loc, Age, Gen, Chron, SupIns,Cont, Dens, Mar, FatherCont

/STATISTICS = default tol

/CRITERIA = TOLERANCE (0.01)

/DEPENDENT = IndPrev

/METHOD = ENTER

/SAVE = RESID

/RESIDUALS.

*___Running ordered probit regression for GP without the index of PHB factors __.

PLUM

VisGP WITH IndPrev, SES, Loc, Age, Gen, Chron, SupIns,Cont, Dens

/LOCATION

/CRITERIA = CIN(95) DELTA(0.0) LCONVERGE(0) MXITER(200) MXSTEP(10) PCONVERGE(0)

/LINK = PROBIT

/MISSING=INCLUDE

/PRINT = FIT PARAMETER SUMMARY TPARALLEL.

*___Running ordered probit regression for GP with the index of PHB factors __.

PLUM

VisGP WITH IndPrev, SES, Loc, Age, Gen, Chron, SupIns, Cont, Dens, RESID

/LOCATION

/CRITERIA = CIN(95) DELTA(0.0) LCONVERGE(0) MXITER(200) MXSTEP(10) PCONVERGE(0)

/LINK = PROBIT

/MISSING=INCLUDE

/PRINT = FIT PARAMETER SUMMARY TPARALLEL.

*___Running ordered probit regression for SD (without the index of PHB factors as *___explained in the article)__.

PLUM

VisSD WITH IndPrev, SES, Loc, Age, Gen, Chron, SupIns,Cont, Dens

/LOCATION

/CRITERIA = CIN(95) DELTA(0.0) LCONVERGE(0) MXITER(200) MXSTEP(10) PCONVERGE(0)

/LINK = PROBIT

/MISSING=INCLUDE

/PRINT = FIT PARAMETER SUMMARY TPARALLEL.

EXECUTE.
